# Understanding the Matrix: The Role of Extracellular DNA in Oral Biofilms

**DOI:** 10.3389/froh.2021.640129

**Published:** 2021-03-22

**Authors:** Hannah J. Serrage, Mark A. Jepson, Nadia Rostami, Nicholas S. Jakubovics, Angela H. Nobbs

**Affiliations:** ^1^Bristol Dental School, University of Bristol, Bristol, United Kingdom; ^2^Wolfson Bioimaging Facility, University of Bristol, Bristol, United Kingdom; ^3^School of Dental Sciences, Newcastle University, Newcastle upon Tyne, United Kingdom

**Keywords:** eDNA, plaque, biofilm, periodontitis, caries, extracellular matrices

## Abstract

Dental plaque is the key etiological agent in caries formation and the development of the prevalent chronic oral inflammatory disease, periodontitis. The dental plaque biofilm comprises a diverse range of microbial species encased within a rich extracellular matrix, of which extracellular DNA (eDNA) has been identified as an important component. The molecular mechanisms of eDNA release and the structure of eDNA have yet to be fully characterized. Nonetheless, key functions that have been proposed for eDNA include maintaining biofilm structural integrity, initiating adhesion to dental surfaces, acting as a nutrient source, and facilitating horizontal gene transfer. Thus, eDNA is a potential therapeutic target for the management of oral disease–associated biofilm. This review aims to summarize advances in the understanding of the mechanisms of eDNA release from oral microorganisms and in the methods of eDNA detection and quantification within oral biofilms.

## Introduction

The oral cavity is home to a diverse range of microbes from at least 2,000 taxa, with ~100 species residing in an individual's mouth at any given time [1, 2]. Those microbes that colonize the teeth form the dental plaque biofilm, which is the key etiological agent in oral diseases such as caries and periodontitis. Members of the plaque microbiota are also associated with the onset of systemic diseases, including Alzheimer's disease [3], rheumatoid arthritis [4], atherosclerosis [5], and infective endocarditis [6]. Thus, therapeutic strategies that can modulate plaque biofilm development and composition have potential to promote both oral and systemic health.

Dental plaque biofilm formation is initiated via the complex interactions of primary colonizers such as *Streptococcus* and *Actinomyces* species with the salivary pellicle that naturally forms on the tooth surface. In turn, these species promote the acquisition of secondary colonizers, which include bridging organisms such as *Fusobacterium nucleatum* and *Corynebacterium* species, together with pathobionts such as *Porphyromonas gingivalis* [7, 8]. As biofilm formation progresses, bacterial species produce and encase themselves within a rich, extracellular matrix known as the extracellular polymeric substance (EPS). This accounts for >90% of biofilm dry weight [9] and comprises a network of molecules that can include polysaccharides, lipids, proteins, and extracellular DNA (eDNA).

Labeling of double-stranded DNA (dsDNA) by immunofluorescence or using DNA dyes such as YOYO-1 has been used to provide evidence of eDNA in biofilms derived from dental plaque samples [10, 11]. Furthermore, application of DNase has been shown to induce significant reductions in early (<8 h) plaque biofilm formation [11]. Such studies are indicative of the critical role of eDNA in oral biofilms, but also highlight eDNA as an attractive target for the management of plaque biofilm formation. This review aims to summarize current understanding of eDNA within dental plaque biofilms, to highlight the tools that can be exploited to detect and quantify eDNA and to provide a perspective on the knowledge gaps that need to be addressed for therapeutic targeting of eDNA to become a reality.

## The Role of eDNA in Oral Biofilms

Initial studies evaluating the role of eDNA in oral biofilm formation revealed that DNase was effective in disrupting biofilm formation of both mixed and single species oral biofilms [12], implying a role for eDNA in maintaining biofilm structural integrity. This was further supported by studies focusing on caries-associated bacterial species *Streptococcus mutans* [13, 14]. Addition of DNA to biofilms containing *S. mutans* increased aggregation in a DNA-dependent manner, improved strength of hydrogen bonds between bacteria, and neutralized repulsive van der Waals forces and acid–base interactions [15]. eDNA was also shown to enhance adhesion of *S. mutans* to hydrophobic surfaces and increased the viscoelasticity of the biofilm [16, 17].

Perhaps linked to a structural role, there is evidence that eDNA may be particularly important in early biofilm formation. For early (<4 h) *Enterococcus faecalis* biofilms, in conjunction with cell wall–anchored proteins PrgB and PrgA, eDNA was shown to promote binding to abiotic surfaces [18]. The PrgB adhesin domain binds DNA *in vitro*, which promotes establishment of early biofilms [19, 20]. eDNA levels within *S. mutans* biofilms increase in a time-dependent manner throughout exponential phase of growth to a maximum 5 h postinoculation [21]. Correlating with this, application of DNase was effective in reducing biofilm formation only up to 8 h postinoculation and had little effect following further incubation [11]. It is hypothesized that as biofilm formation progresses, eDNA may be protected from enzymatic digestion by other macromolecules or potentially replaced by alternative matrix components [22]. Nonetheless, DNase application has been found to weaken the mature biofilm matrix, an effect that may improve the ability of adjunctive antimicrobial agents to penetrate the biofilm [12].

eDNA also acts as a potential reservoir for horizontal gene transfer. Application of eDNA purified from *Veillonella dispar* was capable of transferring tetracycline resistance to *Streptococcus mitis* [23]. Similarly, hydrogen peroxide (H_2_O_2_)–induced eDNA release from *Streptococcus gordonii* led to increased competence of the microbial cell population and thus an increased capability for the uptake of antibiotic resistance traits [24]. Alongside promoting spread of antimicrobial resistance, eDNA has potential to provide protection against cationic antimicrobial peptides and can act as a shield against aminoglycoside antibiotics because of its partial negative charge [25].

Because of its abundance in carbon, nitrogen, and phosphorous, eDNA can function as a nutrient source [discussed by Vorkapic et al. [26]]. In phosphate-limiting environments, *Pseudomonas aeruginosa* (associated with endodontic infection) has been shown to release extracellular DNases, enabling the use of eDNA as a nutrient source [27]. The secretin HofQ, expressed by oral pathogen *Aggregatibacter actinomycetemcomitans* in nutrient-limiting environments, has also been associated with DNA uptake [28].

In relation to oral disease, it has been proposed that eDNA may exacerbate oral inflammation. eDNA isolated from bacterial species associated with endodontic infection (*E. faecalis* and *P. aeruginosa*) induced a low-grade inflammatory response from macrophages [29]. However, further work is required to fully evaluate the role of eDNA directly in modulating oral inflammation vs. other molecules that copurify with eDNA as part of the biofilm matrix.

## eDNA Release Mechanisms

Studies elucidating the molecular mechanisms of eDNA release within oral biofilms have predominantly used monospecies, *in vitro* biofilm cultures. The main focus to date has been on primary colonizers, in particular members of the *Streptococcus* genus, but there is some evidence regarding the mechanism of eDNA release for pathobionts, including *A. actinomycetemcomitans*.

### *Streptococcus mutans* 

**Figure 1:**
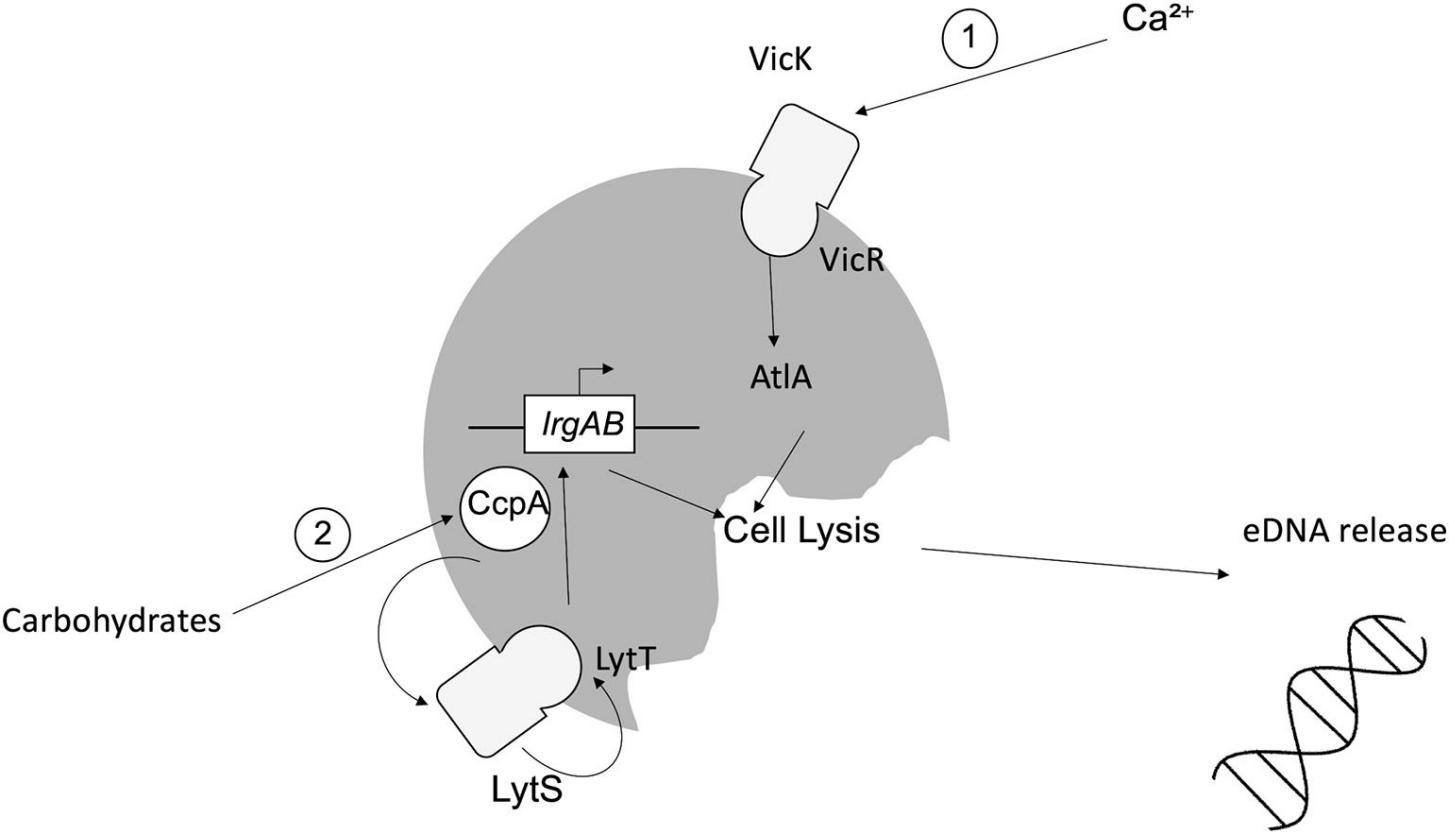
Mechanisms of lysis-dependent eDNA release from *Streptococcus mutans*. (1) Fluctuations in the availability of calcium induce the activity of the sensor kinase VicK, which in turn induces the activity of intracellular response regulator VicR via phosphorylation, leading to the induction of AtlA, autolysis and eDNA release. (2) Carbohydrates (e.g., sucrose/glucose) induce the activity of CcpA, which modulates TCS LytST, ultimately causing the upregulation of *lrgAB* and subsequent expression of the LrgA/B holin-like proteins, inducing cell lysis and eDNA release.

Studies to date suggest that eDNA release from *S. mutans* can be mediated by extrusion of membrane vesicles or through (auto)lysis [15]. For the latter, murein hydrolase autolysin A (AtlA) has been implicated, the activity of which is calcium dependent. Calcium modulates the two-component system (TCS) VicRK, which in turn alters the N-terminus of AtlA, enabling AtlA to induce cell lysis and thus increased eDNA release [30]. A second TCS, LytST, is also implicated in lysis-dependent eDNA release. LytST regulates expression of *lrgAB* and thus transcription of the LrgA/B holin-like proteins, inducing degradation of the host cell wall and eDNA release [21, 31]. LytST is also governed by catabolite control protein A (CcpA), which detects fluctuations in the availability of carbohydrates, including sucrose and glucose [32–34]. These mechanisms are summarized in Figure 1. Additional lysis-dependent eDNA release mechanisms identified in *S. mutans* involve competence-stimulating peptide (CSP) and competence-inducing peptide. The actions of both peptides culminate in the activation of effector ComX, leading to upregulation of autolysis effectors CipB and mutacin V, leading to eDNA release [35–37].

Release of eDNA via membrane vesicles is thought to be lysis independent. Membrane vesicle production has been associated with transpeptidase sortase A (SrtA) as, in the absence of SrtA, *S. mutans* biofilms exhibit reduced protein content within membrane vesicles [21]. However, while eDNA-containing membrane vesicles have been observed within *S. mutans* biofilms [21], the mechanism of active vesicle release into the extracellular environment has yet to be elucidated.

### *Streptococcus gordonii* 

**Figure 2:**
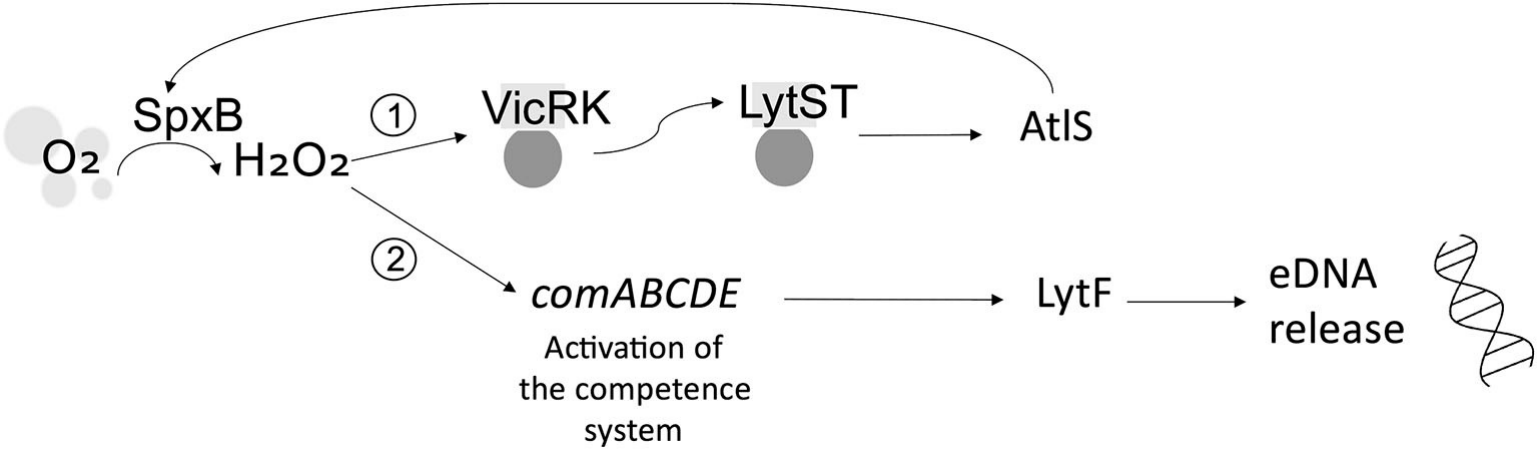
Possible mechanisms of eDNA release from *Streptococcus gordonii*. Under aerobic conditions, pyruvate oxidase (SpxB) converts oxygen (O_2_) to hydrogen peroxide (H_2_O_2_). (1) Indirect: H_2_O_2_ induces TCS VicRK, which, in turn, induces TCS LytST and activation of autolysin AtlS, which feeds back to modulate SpxB and the H_2_O_2_-dependent activity of a second pathway. (2) Direct: H_2_O_2_ induces the competence system (*comABCDE*), mediating activation of LytF and resultant eDNA release.

*Streptococcus gordonii* is a pioneer colonizer of the oral cavity and ubiquitous constituent of dental plaque. As such, *S. gordonii* plays an important role in initiating the accretion of dental plaque on the salivary pellicle and can influence whether the microbial community that develops is predisposed to oral health or disease [38]. *S. gordonii* eDNA is hypothesized to be of chromosomal origin, and its release is associated with H_2_O_2_ [24, 39]. Initial studies revealed that, under aerobic conditions, pyruvate oxidase (SpxB) catalyzes the conversion of oxygen to H_2_O_2_, which activates TCS VicRK. This, in turn, modulates a second TCS, LytST, which regulates the activity of autolysin AtlS [40]. LytT binds the promoter of AtlS to induce eDNA release [40]. AtlS has been shown to have ~30% homology with AtlA, the autolysin associated with eDNA release by *S. mutans* [41]. However, it was subsequently inferred that AtlS is not directly involved in eDNA release within *S. gordonii* biofilms. Rather, following induction, AtlS feeds back to modulate the activity of SpxB and thus intracellular levels of H_2_O_2_. H_2_O_2_ then induces the activity of murein hydrolase LytF, which is absolutely required for eDNA release [41]. LytF is regulated by the *comABCDE* competence operon, which controls DNA uptake by *S. gordonii* [42]. The competence system is inducible by H_2_O_2_. As for *S. mutans*, CSP induces eDNA release in *S. gordonii* biofilms [43]. Specifically, LytF is proposed to induce eDNA release either via lysis of a subpopulation of the microbial cell population or through incomplete bacterial cell lysis, in which the cell envelope remains largely intact [41]. These proposed mechanisms are illustrated in Figure 2.

### *Streptococcus sanguinis* 

*Streptococcus sanguinis* is another primary colonizer of the oral cavity, and alike to *S. gordonii*, the mechanism of eDNA release has been shown to be H_2_O_2_ dependent. Nonetheless, there are conflicting reports as to whether or not the H_2_O_2_-dependent release of eDNA is observed with or without observable cell lysis [39, 44]. Regardless, as for *S. gordonii*, pyruvate oxidase (Spx) is pivotal in modulating the release of eDNA in *S. sanguinis* biofilms via the murein hydrolase LytF [45] and also the catabolite control protein CcpA [46]. Where lysis has been associated with eDNA release, it has been shown that application of either endogenous or exogenous H_2_O_2_ induces apoptosis, accompanied by DNA fragmentation and membrane potential depolarization. A link among H_2_O_2_, the competence system, and eDNA release was also implicated [44].

### *Enterococcus faecalis* 

*Enterococcus faecalis* is commonly found in endodontic infections [47]. *E. faecalis* biofilm formation has been shown to be DNA-dependent, and both lysis-dependent and -independent mechanisms have been described. The first studies revealed that eDNA release from late (>8 h) *E. faecalis* biofilms can be mediated by a fratricidal mechanism of autolysis, in which a subpopulation of cells within the biofilm is targeted for autolysis and thus eDNA release [48]. Gelatinase (GelE) and serine protease (SprE) were implicated in this fratricidal release of eDNA [49]. GelE induces eDNA release, whereas SprE prevents lysis, with both acting by regulating the activity of autolysin Atn. By contrast, for early (<4 h) biofilms, there is no observable cell lysis but rather an elevated membrane potential indicative of a possible energy requirement for active DNA secretion [48–50]. This is proposed to involve the conjugation apparatus, specifically the matrix channel encoded on plasmid pCF10 [50].

### *Aggregatibacter actinomycetemcomitans* 

eDNA has also been isolated from biofilms of *A. actinomycetemcomitans*, a facultative anaerobe commonly associated with the progression of periodontitis. One protein that has been associated with eDNA release from *A. actinomycetemcomitans* is the secretin HofQ, deletion of which results in decreased eDNA release in the presence of cytokines [28].

## Structure of eDNA

Attempts have been made to observe the structure of eDNA in plaque biofilm using high-resolution scanning electron microscopy (SEM). Using an anti-dsDNA primary antibody followed by a 10-nm gold conjugate secondary antibody, eDNA was visualized as a “lattice-” or “net-like” structure comprising strands of eDNA [10, 51]. Similar lattice-like networks have been reported for biofilms of individual oral bacteria, such as *S. gordonii* [43]. This conforms to the “electrostatic net” model for eDNA, which proposes that in low pH environments, negatively charged eDNA forms electrostatic interactions with positively charged cell membrane–anchored lipoproteins and/or DNA-binding proteins within the biofilm EPS, acting as a net that interconnects the cells [52–54].

**Figure 3:**
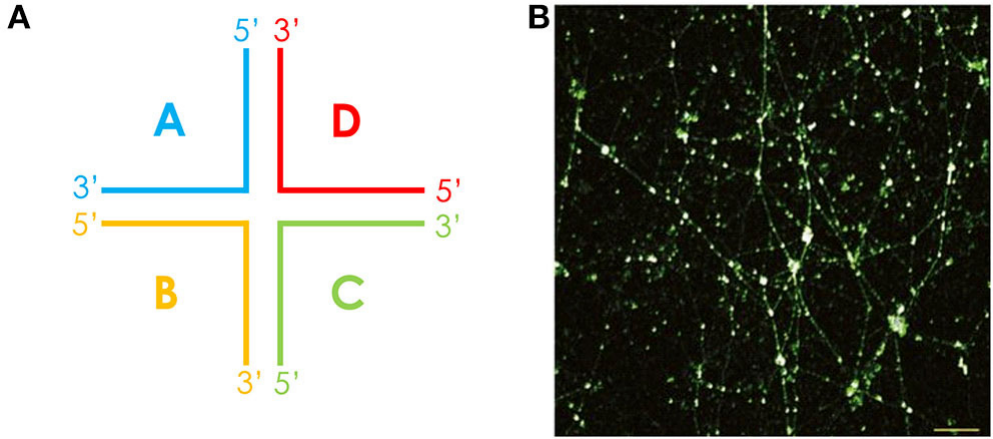
The eDNA lattice within biofilms is structurally related to a Holliday junction. **(A)** The cruciform-like structure of a Holliday junction, consisting of four double-stranded DNA arms that are joined. The Holliday junction may also adopt alternative conformations. **(B)** These structures were first observed in a chinchilla middle ear infected with NTHI, where cruciform DNA structures were stained in white and dsDNA in green. Scale bar, 10 μm. Reproduced from [55].

For many oral bacteria, histone-like proteins have been associated with the eDNA network within biofilms. For non-typeable *Haemophilus influenzae* (NTHI), it was found that the strands of eDNA displayed a Holliday junction–like configuration in combination with DNA-binding proteins such as integration host factor and histone-like protein (HU), which served to stabilize these junctions [55] (Figure 3). Likewise, HU was reported within *S. gordonii* biofilms, and antiserum directed against this protein was shown to disrupt biofilm stability. This antiserum also disrupted *S. mitis* and *Streptococcus oralis* biofilms, reflecting the high levels of homology seen for HU across these bacteria [57]. By contrast, the *P. gingivalis* eDNA matrix is stabilized by histone-like protein-β (HUβ), which is antigenically distinct from HU [58]. Exploiting this, Rocco et al. demonstrated that targeting HUβ could specifically prevent the acquisition of *P. gingivalis* into preformed biofilms of *Streptococcus* species, including *S. oralis, S. mitis*, and *S. gordonii* [58].

For *E. faecalis*, the structure of eDNA has been shown to vary in a time-dependent manner. In early (4 h) biofilms, eDNA structures were described as “yarn-like,” appearing as interwoven threads across the biofilm. By contrast, in late (8 h) biofilms, eDNA appeared as a “sweater-like” structure, appearing more as a mat of eDNA. In both instances, however, lattice-like and cruciform structures could still be observed, particularly in early biofilms, providing further support for the notion that eDNA forms Holliday junction–like structures with protein within oral biofilms [50].

Alongside histone-like proteins, for *S. mutans*, there is evidence that glucans may support eDNA within the biofilm matrix. Glucans and eDNA have been shown to colocalize within biofilms [59], and in the presence of sucrose, levels of eDNA are elevated [60]. However, even in the absence of glucosyltransferases, eDNA remains abundant within *S. mutans* biofilms, indicating that the eDNA network is not glucan dependent [59, 61]. In addition, isolation of eDNA from *P. aeruginosa* biofilm using solid-state analysis revealed eDNA can adopt unusual structures including non-canonical Hoogsteen base pairs, triads, and tetrads [62]. It remains to be seen whether these are present or important in oral biofilms.

## Methods for the Detection and Quantification of eDNA

To fully elucidate the role of eDNA within oral biofilms, reliable, and reproducible methods of eDNA detection and quantification are required. To date, the majority of studies have utilized extraction of soluble DNA from either monospecies or multispecies biofilms followed by DNA quantification methods including PicoGreen assay, UV-Vis spectrometry or semiquantitative polymerase chain reaction (PCR) (Table 1). These methods allow direct assessment of eDNA within biofilms, and using real-time PCR with species-specific (often 16S rRNA) primers, it can be possible to determine the relative levels of eDNA released from different bacterial strains within mixed species biofilms (Table 1). Nonetheless, a limitation of these quantification approaches is that they provide no information regarding the structural composition or architecture of the eDNA lattice-like networks. Likewise, studies evaluating the capacity for DNase application to impair oral biofilm formation infer the presence of eDNA but provide no direct information regarding the nature of the eDNA network [66, 70, 71]. Rather, to assess these parameters, microscopy approaches are required (Table 1). Two studies have found evidence of the “lattice-” or “net-like” structures of eDNA in dental plaque and dual species (*S. gordonii* and *Candida albicans*) biofilms by confocal microscopy using dsDNA-specific antibodies conjugated to a fluorescent secondary antibody [10, 43]. However, using nucleic acid stain SYTO9, Rainey et al. [59] were not able to visualize networks of eDNA in 16 h *S. mutans* biofilm. This may have been due to the time point at which eDNA release was evaluated, as eDNA release is proposed to be at its maximum in *S. mutans* biofilm at 5 h postinoculation [21]. Alternatively, it may be due to a limitation of SYTO9, which is commonly used to stain intact bacterial cells rather than extrusions of DNA. Thus, it seems that anti-dsDNA conjugate antibodies or TOTO-1 are most efficacious for the reliable detection of eDNA using fluorescence microscopy. Nonetheless, further work is required to optimize these imaging tools for not only the reliable visualization of eDNA within oral biofilms but also its quantification. SEM can also be used to assess eDNA structures at high resolution [66]. However, without the application of a DNA-specific antibody prior to imaging, it is difficult to ascertain whether structures observed are indeed eDNA or alternative ECM components [50]. Alternatively, extraction with ionic liquids also preserves the structure of eDNA in *P. aeruginosa* biofilm, providing a method for elucidating the intricacies of eDNA structure and its interactions with nucleic acids including RNA [62]. Going forward, an important consideration will be how to ascertain the relative contributions of different species within an oral biofilm consortium to total eDNA release. It may be possible to combine the fluorescent labeling of dsDNA with techniques such as fluorescence *in situ* hybridization (FISH), which enables differential labeling of microbes by use of fluorescent probes that bind to genus- or species-specific nucleic acid sequences. For example, CLASI-FISH has been successfully used to investigate the biogeography of dental plaque samples [8]. A combination of these microscopy approaches could help to identify the origin and dynamic of eDNA release within polymicrobial communities.

**Table 1:** Methods for detection and quantification of eDNA within oral biofilms.

**Experimental approach**	**Analysis method**	**References**
DNA extraction	UV-Vis spectrometry	[21, 43, 61, 63–65]
	Semiquantitative PCR	[30, 45, 66, 67]
	Fluorescence-based assay	[21, 28, 68, 69]
	Real-time PCR	[33, 39, 41, 46, 59, 68]
DNase application	Biomass evaluation	[66, 70, 71]
Microscopy	Confocal laser scanning microscopy^*^	[10, 11, 43, 57–59, 66]
	Scanning electron microscopy^*^	[66]

* *Technique evaluates the structural features of eDNA rather than direct quantification of eDNA within biofilms*.

## eDNA as a Therapeutic Target

With growing evidence for the role of eDNA in maintaining oral biofilm structural integrity, eDNA is an attractive potential target for modulating dental plaque formation. One way in which this might be achieved is via application of DNase using toothpaste or mouthwash as a delivery system [discussed by Jakubovics and Burgess [12]]. Bacterial-specific enzymes such as NucB isolated from *Bacillus licheniformis* have shown efficacy in inhibiting the formation of biofilms produced by oral streptococci, including *Streptococcus intermedius* and *Streptococcus salivarius* [72]. Application of NucB to plaque biofilm derived from saliva samples significantly reduced microbial load (evaluated by next-generation sequencing) and modulated biofilm composition. The relative proportion of health-related genera, including *Streptococcus* and *Veillonella* species increased following NucB treatment, whereas *Porphyromonas* and *Prevotella* species were reduced [10]. These studies indicated a species-specific response to DNase enzymes. This characteristic may prove particularly useful in modulating oral biofilm formation, for which the end goal is to promote a microbial community in homeostasis with the host rather than biofilm eradication. Coating of surfaces could also prove a novel delivery system for DNase. Coating of polymethylmethacrylate, a material commonly used in dental restorations, with DNase I, has been shown to prevent colonization by *Staphylococcus aureus* and *P. aeruginosa* [73]. Nonetheless, further work is required to understand the wider spectrum of species-specific responses of oral bacteria to DNases and to characterize DNase enzymes that can exert a desirable modulatory effect on biofilm communities to promote oral health when delivered individually or in combination.

DNase enzymes might also be used as an adjunct to enhance the penetration and thus efficacy of other antibiofilm treatments, such as antibiotics [74]. This approach has also shown promise for antimicrobial photodynamic therapy (aPDT). Treatment of biofilms with DNase I prior to use of aPDT increased biofilm dispersal relative to aPDT alone due to improved ability of the photosensitizer to penetrate the biofilm [75]. Of note, blue light has proven efficacious in inhibiting the formation of *F. nucleatum* and *P. gingivalis* biofilm [56]. As blue light (400–450 nm) is close to the UV section of the electromagnetic spectrum, it is possible that one explanation for its antibiofilm effects is the induction of eDNA damage.

Alongside exploitation of DNase enzymes, another strategy could be to target the DNA-binding proteins that associate with eDNA [76]. As discussed, antiserum against *P. gingivalis* HUβ prevented acquisition of this pathobiont into a preformed oral biofilm [58], whereas antiserum against *S. gordonii* HU disrupted biofilm formation by not only *S. gordonii* but also *S. mitis* and *S. oralis* [57]. Removal of HU proteins has also been found to increase the porosity of biofilms, indicating that an adjunctive role to make biofilms more accessible to antimicrobial agents may be possible [54]. Further work will be required to determine, for example, the cost efficiency of generating HU antisera and their compatibility with being incorporated into products such as mouthwash or toothpaste.

Natural products have been shown to inhibit eDNA-dependent biofilm formation. Derived from the adzuki bean, 7S globulin 3 and theaflavin-3,3′-digallate inhibited biofilm formation by *S. mutans* via modulation of eDNA production [67, 77]. The synthesized helical peptide, G3, has also shown efficacy in modulating *S. mutans* eDNA-dependent biofilm formation. G3 binds and thus destabilizes the eDNA matrix, inducing biofilm dispersal [78]. Sodium hypochlorite, an antiseptic used in endodontic treatments, can impair eDNA-dependent biofilm formation by *E. faecalis* [66]. However, it is not yet known how such compounds would function in dispersal of the complex biofilm communities found within the oral cavity.

## Conclusions

There is strong evidence to suggest that eDNA is an integral component of dental plaque–associated biofilm, with potential to be targeted for the therapeutic manipulation of plaque development to promote oral health. Nonetheless, for such strategies to become a reality and translated into commercial products, a much greater understanding of eDNA architecture and release is required. For some oral bacteria, there is evidence that the mechanism of eDNA release can involve autolysins and the competence system. However, further work is required to fully elucidate not only the molecular basis of eDNA release from these monospecies oral biofilms, but also how interactions between microbes within the complex plaque communities *in vivo* may further modulate eDNA release. Effective targeting of eDNA will also require improved insight into the molecules with which eDNA complexes to form its characteristic lattice-like networks. A combination of advanced imaging and quantification techniques, used in conjunction with appropriate oral biofilm models, will be needed to achieve such objectives.

## Author Contributions

HS: contributed to conception, design, drafted and critically revised the manuscript. MJ: critically revised the manuscript. NJ and NR: contributed to conception and critically revised the manuscript. AN: contributed to conception, design and critically revised the manuscript. All authors gave their final approval and agree to be accountable for all aspects of the work.

## Conflict of Interest

The authors declare that this work was conducted in the absence of any commercial or financial relationships that could be construed as a potential conflict of interest.
